# OSMR coordinates a self-perpetuating circuit linking chemoresistance and neutrophil-driven immunosuppression in gastric cancer

**DOI:** 10.1016/j.neo.2026.101279

**Published:** 2026-02-06

**Authors:** Dan Wei, Jiang Chen, Feihu Bai, Donglin Li, Jie Liu, Hongjia Dou, Yan Lei, Yongzhen Zhang, Bo Zhang, Ying Pang, Changchun Cao, Tao Yang, Junling Han, Tianyu Cao

**Affiliations:** a Department of Hepatobiliary Surgery, Xijing Hospital, Fourth Military Medical University, Xi'an, China; b The First Affiliated Hospital of Guizhou University of Traditional Chinese Medicine, Guiyang, China; c Department of Gastroenterology, The Second Affiliated Hospital of Hainan Medical University, Haikou, China; d The Armed Police Corps Hospital of Xinjiang Uygur Autonomous Region, Urumqi, China; e The 928 Hospital, Haikou, China; f Suqian People's Hospital affiliated to Nanjing Drama Tower Hospital Group, Suqian, China; g State Key Laboratory of Holistic Integrative Management of Gastrointestinal Cancers and Department of Biochemistry and Molecular Biology, Fourth Military Medical University, Xi’an 710032, China

**Keywords:** OSMR, Chemoresistance, Gastric cancer, BMP5, IL31

## Abstract

Chemoresistance and immunosuppression present major challenges in gastric cancer (GC) treatment, with their interplay remaining poorly understood. We identify the Oncostatin M receptor (OSMR) as a central regulator coordinating both chemoresistance and neutrophil-mediated immunosuppression. OSMR was significantly upregulated in GC patients, correlating with poor chemotherapy response and reduced CD8^+^*T* cell infiltration. Mechanistically, OSMR directly recruits PI3K, amplifying PI3K/AKT signaling to increase cyclin E2 (CCNE2) expression, thereby sustaining tumor cell survival under chemotherapy-induced stress. Crucially, we uncovered a novel immunoregulatory cascade: OSMR drives BMP5 transcriptional activation, orchestrating N2-polarization of tumor-associated neutrophils (TANs) and upregulating PD-L1 expression on TANs, ultimately impairing CD8^+^*T* cell cytotoxicity. Dysfunctional CD8^+^*T* cells secreted IL31, activating the OSMR pathway in GC cells and thereby forming a self-perpetuating OSMR-BMP5-IL31 feedback circuit that sustains therapeutic resistance. Therapeutically, OSMR neutralization with vixarelimab synergized with fluorouracil to overcome chemoresistance and reinstate anti-tumor immunity in GC preclinical models. Our findings establish OSMR as a molecular linchpin connecting intrinsic tumor survival pathways (PI3K/CCNE2) with extrinsic immunosuppressive reprogramming (BMP5/TANs/CD8^+^*T* cells), providing a clinically actionable target to overcome treatment resistance in GC.

## Introduction

Gastric cancer (GC) ranks as the fifth most common malignancy and the fourth leading cause of cancer-related deaths globally, with over 1 million new cases and 769,000 deaths annually [1]. Despite improvements in surgery and chemotherapy, the 5-year survival rate for advanced GC remains under 30% [2]. Additionally, over 50-60% of patients experience disease recurrence within five years of resection, with median survival dropping to <12 months after relapse [3]. The poor prognosis and high recurrence rate in GC patients are mainly attributed to chemoresistance [4]. While the initial success of early chemotherapy generates enthusiasm among patients, this optimism is often short-lived. Tumors may rapidly enter remission, but they inevitably develop chemoresistance, leading to disease recurrence [5]. The mechanisms underlying this chemoresistance are diverse, encompassing factors such as tumor heterogeneity, tumor burden, and the tumor microenvironment [6]. Our previous research demonstrated that GC cells secreted CGA to activate EGFR, establishing a positive feedback loop that enhanced chemoresistance [7]. The complexities of chemoresistance remain only partially understood, underscoring the necessity to elucidate the molecular mechanisms underlying chemoresistance and to identify viable therapeutic strategies.

Recent studies highlighted the critical role of the immunosuppressive tumor microenvironment (TME) in the recurrence of GC, which is characterized by dysregulation of immune cell infiltration [8]. Neutrophils, the most abundant white blood cells in the human body, demonstrate significant phenotypic plasticity and functional heterogeneity within the microenvironment of solid tumors [9]. Their roles have evolved from serving primarily as the initial line of defense in the innate immune system to becoming essential components of the tumor immune regulatory network [10,11]. Tumor-associated neutrophils (TANs) exhibit dual roles in tumor dynamics, functioning both as stimulators and inhibitors, and can be classified into two distinct subtypes: N1 and N2 [12]. Each subtype is characterized by unique immunological profiles and functions. N1 TANs are marked by elevated levels of CCL3, TNF, and ICAM-1, alongside diminished arginase activity. Conversely, N2 TANs are characterized by high expression of various CCL and CXCL chemokines, which may influence cancer progression through the modulation of T cell activity [13,14]. Previous research indicates that neutrophils with increased expression of PD-L1 can suppress immune responses by inducing T-lymphocyte apoptosis and promoting immunosuppression [15]. Interventions targeting PD-L1 in neutrophils, through the application of DNAse or anti-PD-L1, have demonstrated a reduction in tumor growth, suggesting a novel approach to preserving immune function within the TME [16]. Nevertheless, the role and underlying mechanism of neutrophils in the chemoresistance of GC warrant further investigation.

The Oncostatin M receptor (OSMR), a constituent of the interleukin-6 receptor family, functions as a common receptor for oncostatin M (OSM) and interleukin-31 (IL31), facilitating signal transduction essential for processes such as inflammation, metastasis, and immune regulation [17,18]. Overexpression of OSMR was associated with unfavorable prognoses in various cancers, such as pancreatic ductal adenocarcinoma and ovarian cancer [19,20]. OSMR was found to facilitate cellular proliferation, invasion, and chemoresistance by activating STAT5a, STAT5b, Akt, JNK, P38, and PKCd, with these effects being context-dependent [21,22]. In GC, OSMR was upregulated in recurrent tumors and is correlated with advanced TNM staging and lymph node metastasis [23]. Except from its oncogenic functions, OSMR influenced the TME by inducing macrophage polarization towards an immunosuppressive M2 phenotype and inhibiting cytotoxic T-cell activity [24]. Vixarelimab, a humanized monoclonal antibody targeting OSMR, has been awarded Breakthrough Therapy Designation by the United States Food and Drug Administration (FDA) for the treatment of pruritus associated with nodular prurigo [25,26]. The antibody have demonstrated a rapid reduction in itch intensity and significant improvement in skin lesions in two Phase II randomized controlled trials (NCT03816891, NCT03858634), while maintaining a favorable safety profile [27]. Collectively, the findings from these OSMR-related oncology studies indicated an initial association between OSMR and immunosuppression and pro-tumor activity, but the direct correlation between the OSMR and GC chemoresistance remains to be established.

In this study, we systematically analyzed the multifaceted roles of OSMR in promoting GC chemoresistance and immunosuppression. Our findings indicated that OSMR overexpression in GC tissues induced chemoresistance through activation of PI3K-AKT signaling pathway and elevation of CCNE2. Additionally, we demonstrated that OSMR overexpression led to the downregulation of TRIM2, an E3 ligase, which contributed to decreased ubiquitination of GATA3 and increased expression of BMP5. BMP5 was then secreted into the TME by tumor cells, causing N2 polarization of neutrophils and transcriptional expression of PD-L1. High expression of PD-L1 in neutrophils inhibited the function of CD8^+^ *T* cells and induces their secretion of IL31, which in turn intensifies the activation of OSMR in tumor cells, forming a cross-linked circuit including tumor cells, neutrophils and T cells. The inhibition of OSMR using Vixarelimab resulted in a reduction of the malignant phenotype in GC cells and an enhancement of T-cell-mediated antitumor immunity. Our study elucidates the OSMR signaling pathways, providing preclinical evidence for the potential repurposing of OSMR-targeted therapies to address chemoresistance and immune evasion in GC.

## Materials and Methods

### Cell culture and treatment

GES-1 (RRID:CVCL_EQ22), BGC823 (RRID:CVCL_3360), NCI-N87 (RRID:CVCL_1603), AGS (RRID:CVCL_0139), SNU-1 (RRID:CVCL_0099), SNU-16 (RRID:CVCL_0076), MGC803 (RRID:CVCL_5334), MKN45 (RRID:CVCL_0434), MKN28 (RRID:CVCL_1416), and MFC (RRID:CVCL_5J48) cells were obtained from the China Infrastructure of Cell Line Resources. All cells were maintained and passaged at the State Key Laboratory of Cancer Biology (CBSKL). All cell lines were authenticated by short tandem repeat analysis and were frequently checked for their morphological features and functionalities. AGS, MKN28, and MFC cells were cultured in RPMI 1640 (Invitrogen, USA). Both media were supplemented with 10% fetal bovine serum (FBS; Gibco, USA).

### Clinical samples

GC samples were purchased from OUTDO BIOTECH company composed of 94 primary tumor tissues and 86 adjacent normal tissues. Samples from GC patients who received chemotherapy were collected from the First Affiliated Hospital of Guizhou University of Traditional Chinese Medicine in accordance with institutional ethical guidelines. Patients’ clinical and pathologic characteristics are summarized in Table S1 and S2.

### Western blotting

Protein was extracted from cells or tissues using RIPA lysis buffer (Beyotime, China) supplemented with protease and phosphatase inhibitors (Roche, Switzerland), quantified via BCA assay (Thermo Scientific, USA). Equal amounts of protein (20–50 μg) were separated by 10% SDS-PAGE and transferred onto NC membranes using a semi-dry transfer system (Bio-Rad, USA). The membranes were blocked with 5% non-fat milk in TBST for 1 hour at room temperature, followed by an overnight incubation at 4°C with primary antibodies (OSMR (Abcam, #ab315388), CCNE2 (Abcam, #ab40890), β-actin (CST, #4967), PI3K (P85) (CST, #4292), p-PI3K (p-P85) (Abcam, #ab278545), AKT (CST, #9272), p-AKT (CST, #4060), P27 (CST, #2552), p-P27 (Abcam, #ab62364), FLAG (CST, #14793), BMP5 (Proteintech, #13253-1-AP), Ub (CST, #20326), TRIM2 (Proteintech, #20356-1-AP), GATA3 (CST, #5852), p-SMAD1/5 (CST, #9516), SMAD5 (CST, #12534), PD-L1 (CST, #13684), BATF (CST, #8638), IL-31 (abcam, #ab62579)), which were diluted in accordance with the manufacturer's instructions. After TBST washes, membranes were incubated with HRP-conjugated secondary antibodies for 1 h at room temperature. Protein bands were visualized using enhanced chemiluminescenceand imaged on a ChemiDoc XRS+ System (Bio-Rad, USA).

### RT-qPCR

Total RNA was extracted from cells or tissues using TRIzol reagent (Invitrogen, USA), quantified by NanoDrop 2000 (Thermo Scientific, USA), and reverse-transcribed into cDNA using the PrimeScript RT Master Mix. qPCR reactions were performed in triplicate using TB Green Premix Ex Taq II (Takara, Japan) on a CFX96 Real-Time System (Bio-Rad, USA) with 20 μL reaction volumes containing 1 μL cDNA, 0.2 μM primers, and nuclease-free water. Relative gene expression was calculated by the 2^(−ΔΔCt) method normalized to GAPDH.

### Cell viability

Cell viability was assessed using the Cell Counting Kit-8 (CCK-8; Dojindo, Japan) according to the manufacturer’s protocol. Briefly, AGS and MKN28 cells were seeded in 96-well plates at a density of 5 × 10³ cells/well in 100 μL complete medium and allowed to adhere overnight. Cells were then treated with Fluorouracil or PBS for 72 h. At each time point, 10 μL of CCK-8 reagent was added to each well, followed by incubation at 37°C for 2 h. Absorbance was measured at 450 nm using a Thermo Scientific Varioskan Flash multimode reader. Each condition was tested in triplicate, and experiments were independently repeated three times. Edge wells were filled with sterile PBS to minimize evaporation artifacts.

### Immunohistochemistry (IHC) analysis

Formalin-fixed, paraffin-embedded (FFPE) tissue sections were mounted on slides, baked at 65°C for 2 hours, deparaffinized in xylene, and rehydrated through graded ethanol. Heat-induced antigen retrieval was performed in 10 mM citrate buffer (pH 6.0). Endogenous peroxidase activity was blocked with 3% H₂O₂ for 15 min, followed by blocking with 5% normal goat serum in TBST for 30 min. Samples were incubated overnight at 4°C with primary antibodies (Ki67 (CST, #34330), Cleaved Caspas-3 (CST, #9664)). After TBST washes, HRP-conjugated secondary antibodies were applied for 30 min, and signals were visualized with DAB chromogen (5 min). Slides were counterstained with hematoxylin, dehydrated and cleared in xylene. Staining was quantified using a composite H-score (intensity × percentage of positive cells), with digital imaging performed on a 3D‐Histech Pannoramic‐250 microscope slide‐scanner and Case Viewer software.

### Multiplex immunofluorescence (mIF)

The multiplex immunofluorescence (mIF) experiment was conducted utilizing the AKOYA kit. The detailed procedure was as follows: Slides were baked at 60-65°C for 2 hours, then immersed in xylene and ethanol. Samples were fixed in 10% formalin for 10-20 minutes, heated, and cooled to room temperature. Slides were treated with deionized water, 3% hydrogen peroxide for 10-15 minutes, and a blocking buffer for 10 minutes. A diluted primary antibody was applied and incubated at 25°C for 1 hour, followed by a 10-minute incubation with secondary antibodies and Opal dye. High-temperature antigen retrieval was repeated, and the primary antibody step was repeated. Finally, DAPI staining was conducted, and images of the slides were captured using the Vectra multispectral imaging system, followed by subsequent analysis. The antibodies included OSMR (Abcam, #ab315388), CCNE2 (Abcam, #ab40890), BMP5 (Proteintech, #13253-1-AP), PD-L1 (CST, #13684), CD15 (Abcam, #ab135377), CD8 (Abcam, #ab237709), IL31 (Proteintech, #22859-1-AP).

### Analysis of public datasets

All TCGA data sets were downloaded from the TCGA data portal (https://tcga-data.nci.nih.gov/tcga/). The Kaplan–Meier-plotter database (http://kmplot.com/analysis/), was used to evaluate the effect of OSMR gene expression on patient survival. Patients were separated into two groups based on median expression of OSMR. Kaplan–Meier survival plots were generated and significance by log-rank p value was computed. Logrank P-value < 0.05 was considered as significant.

### RNA-seq and data analysis

Total RNA was extracted from cells using TRIzol reagent (Invitrogen, USA), followed by quality assessment with an Agilent 2100 Bioanalyzer. Stranded mRNA libraries were prepared from 1 μg of total RNA using the TruSeq Stranded mRNA Library Prep Kit (Illumina) with poly-A selection, fragmented to ∼300 bp, and subjected to paired-end sequencing (150 bp) on an Illumina NovaSeq 6000 platform, generating ≥40 million reads per sample. Transcript quantification and differential expression analysis were performed using featureCounts (v2.0.3) and DESeq2 (v1.34.0). Kyoto Encyclopedia of Genes and Genomes (KEGG) were conducted.

### Immunoprecipitation (IP)

Cells were lysed in ice-cold RIPA buffer (Thermo Scientific, USA) supplemented with protease/phosphatase inhibitors. Protein concentrations were quantified via BCA assay (Thermo Scientific, USA), and 500 μg of lysate was pre-cleared with Protein A/G magnetic beads (Sigma-Aldrich) for 1 h at 4°C with constant rotation. Lysates were incubated overnight at 4°C with 2 μg of anti-OSMR antibody or isotype control IgG. Beads were washed with lysis buffer, and boiled with the SDS loading buffer, followed by western blot analysis. Band intensities were quantified using Image Lab (Bio-Rad, USA).

### Flow cytometry analysis

Cells were harvested by trypsinization (for cells) or mechanical dissociation (for primary tissues), filtered through a 70-μm cell strainer, and washed twice with ice-cold PBS containing 2% FBS. For surface marker staining, 1 × 10⁶ cells were incubated with indicated fluorochrome-conjugated antibodies in the dark at 4°C for 30 min. Intracellular staining was conducted using a fixation/permeabilization kit (BD Biosciences), followed by incubation with the appropriate antibodies. For viability assessment, cells were stained with 7-AAD (1:20, BioLegend) prior to analysis. Data were acquired on an SA3800 Spectral Cell Analyzer (Sony Biotechnology) or a Beckman Coulter Epics XL-MCL Flow Cytometer, followed by quantification of target populations using the Expo 32 ADC software or FlowJo.

### Co-culture experiment

In this study, we employed an in vitro CD8^+^ *T* cell, neutrophil and tumor cell co-culture system. Specifically, CD8^+^ *T* cell and neutrophil were isolated from human peripheral blood mononuclear cells (PBMCs) via magnetic-activated cell sorting (MACS) usingCD8^+^ *T* cell and neutrophil isolation kit (STEMCELL, USA). The co-culture experiment involved seeding CD8^+^ *T* cells and neutrophils or tumor cells with neutrophils into a 24-well plate. After a 48-hour incubation period, the co-cultured cells were subsequently analyzed.

### Mouse xenograft tumor model

Mouse aged 6-8 weeks from various strains (BALB/c-nude, 615) were obtained from Beijing Vital River Laboratory Animal Technology or bred in-house. They were kept in pathogen-free conditions and handled according to the Fourth Military Medical University's Animal Care. For the resistance experiment, MKN28 cells, either with shNC or shOSMR, at a concentration of 5 × 10⁶ cells in a 200 μL PBS, were injected into the outer thigh skin of BALB/c-nude mice. MFC cells with shNC or shNMP5 (5 × 10⁶ in 200 μL PBS) were injected into the outer thigh skin of 615 mice. Tumor volume (*V*= length×width²×0.5) was measured every 3 days using digital calipers. When tumors reached ∼100 mm³, the sh-NC and sBMP5 groups were injected intraperitoneally with 20 mg/kg fluorouracil every 3 days. For combination therapy trial, mouse were injected intraperitoneally with fluorouracil and/or Vixarelimab. Following a treatment period of three weeks, the mice were euthanized in accordance with institutional ethical guidelines. Excised tumors were weighed, photographed, and divided for formalin fixation.

### Luciferase reporter assay

The interaction between GATA3 and BMP5, SAMD5 and PD-L1, BATF and IL31 were investigated utilizing a dual-luciferase reporter assay system. The promoter region of BMP5, PD-L1, or IL31, was amplified via polymerase chain reaction (PCR) and subsequently cloned into the pGL3-basic vector. Plasmids encoding the GATA3, SMAD5, or BATF, -driven firefly luciferase reporter and the CMV promoter-driven Renilla luciferase were co-transfected with FLAG-IL31 into HEK293T cells for a duration of 24 hours. Following transfection, the activities of firefly luciferase and Renilla luciferase were quantified using the Dual-Luciferase Reporter Assay System, in accordance with the manufacturer's instructions.

### Statistical analysis

Statistical significance was evaluated employing multiple methodologies, including Student’s t-tests and Fisher’s exact test, to determine the p-value for comparisons between two groups. For experiments involving more than two groups, a one-way or two-way analysis of variance (ANOVA) was employed. Spearman correlation was used for continuous variables. Kaplan-Meier curves analyzed in vivo survival. Data are shown as means ± standard error of the mean (SEM), with analyses performed using Prism 9.0, SPSS or R scripts. Details are in the figure legends. The thresholds for statistical significance employed in this study were delineated as follows: not significant (ns) for p-values greater than 0.05; * for p-values <0.05; ** for p-values <0.01.

## Results

### OSMR is associated with poor prognosis of GC patients and promotes GC chemosistance

**Fig. 1:**
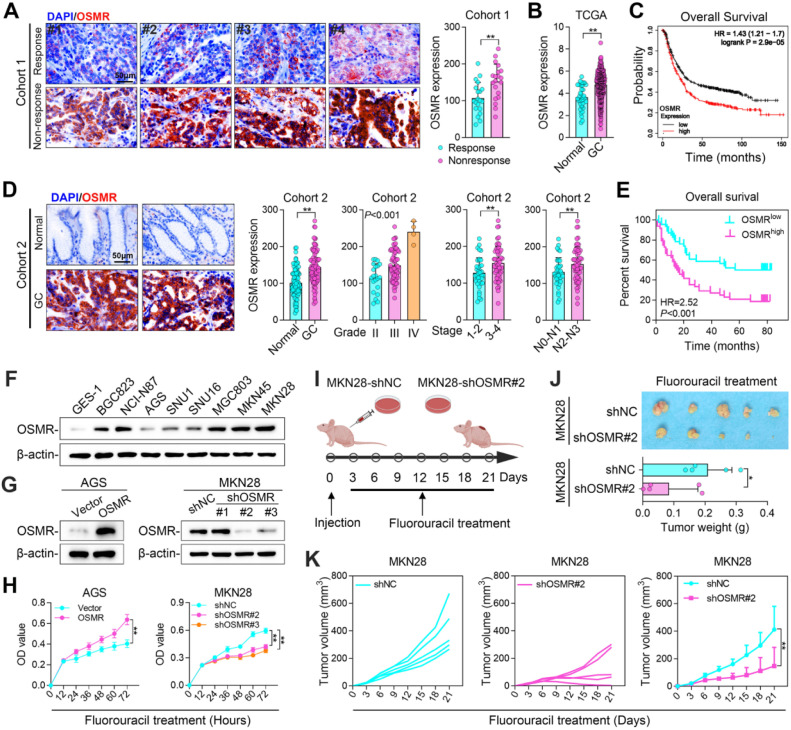
**OSMR is associated with poor prognosis of GC patients and promotes GC chemosistance.** IHC staining of OSMR in tumor samples from patients in Cohort 1. **(B)** TCGA data analysis of OSMR expression in GC and normal stomach tissues. **(C)** Correlation between OMSR expression and GC patients’ overall survival in Kaplan-Meier plotter database. **(D)** IHC staining of OSMR in tumor samples from patients in Cohort 2. **(E)** Survival analysis of correlation between OSMR expression and patients’ overall survival in Cohort 2. **(F)** OSMR protein levels in GC cells. **(G)** OSMR protein levels in AGS and MKN28 cells when OSMR was up-regulated or down-regulated. **(H)** Cell proliferation of AGS and MKN28 cells when OSMR was up-regulated or down-regulated. **(I)** Schematic of an in vivo experiment. **(J)** Photo and weight of tumors from MKN28 cells knocking down OSMR. **(K)** Volume of tumors from MKN28 cells knocking down OSMR. (*, *P* < 0.05, **, *P* < 0.01).

To investigate the role of OSMR in chemoresistance of GC, we collected tumor specimen from 20 patients exhibiting non-responsiveness to chemotherapy and 20 patients demonstrating a positive response to chemotherapy (Cohort 1) (Fig. 1A). The expression of OSMR were assessed using IHC staining techniques. Our findings revealed that OSMR expression was significantly elevated in patients who did not respond to chemotherapy compared to those who did (Fig. 1A). Additionally, analysis of GC database from TCGA indicated that OSMR expression in GC tissues were higher than those in normal gastric tissues (Fig. 1B). Data from the Kaplan-Meier plotter database further indicated that GC patients with elevated OSMR expression experienced poorer Overall Survival (OS), First Progression (FP), and Post-Progression Survival (PPS) (Fig. 1C and S1A). Furthermore, we utilized a GC tissue microarray (Cohort 2), which comprised 86 para-cancerous tissues and 94 GC tissues, to further evaluate OSMR expression (Fig. 1D). The results demonstrated that OSMR expression was significantly higher in tumor tissues compared to adjacent non-cancerous tissues, and it was closely associated with tumor grade, stage and lymph node invasion (Fig. 1D). Prognostic analysis also indicated that patients with high OSMR expression had a worse prognosis (Fig. 1E). These above results suggest that OSMR plays an important role in chemotherapy resistance, grade, stage and poor prognosis of GC.

To investigate the role of OSMR in GC chemoresistance, we conducted an analysis of the mRNA and protein expression levels of OSMR in immortalized gastric epithelial cells (GES-1) and GC cell lines (Fig. 1F and S1B). The findings revealed that OSMR expression was increased in GC cells compared to GES-1 cells. We selected AGS cells, characterized by low OSMR expression, and MKN28 cells, characterized by high OSMR expression, for further experimentation involving the overexpression and knockdown of OSMR (Fig. 1G and S1C). Overexpression of OSMR in AGS cells markedly enhanced their chemoresistance capability, whereas OSMR knockdown in MKN28 cells resulted in a reduction of this ability (Fig. 1H). Subsequently, we inoculated MKN28 cells with reduced OSMR expression into the outer thigh skin of nude mice and administered Fluorouracil by intraperitoneal injection (Fig. 1I). The results demonstrated that OSMR knockdown significantly decreased tumor weight and volume in the presence of the chemotherapeutic agent (Fig. 1J and 1K). IHC analysis using Ki67 and Cleaved Caspase-3 staining indicated that OSMR knockdown diminished both the proliferative and anti-apoptotic capacities of the tumor cells (Figure S1D). These findings suggest that OSMR plays a critical role in enhancing the chemoresistance of GC cells.

### OSMR promotes CCNE2 expression by interacting with PI3K to enhance GC chemoresistance

**Fig. 2:**
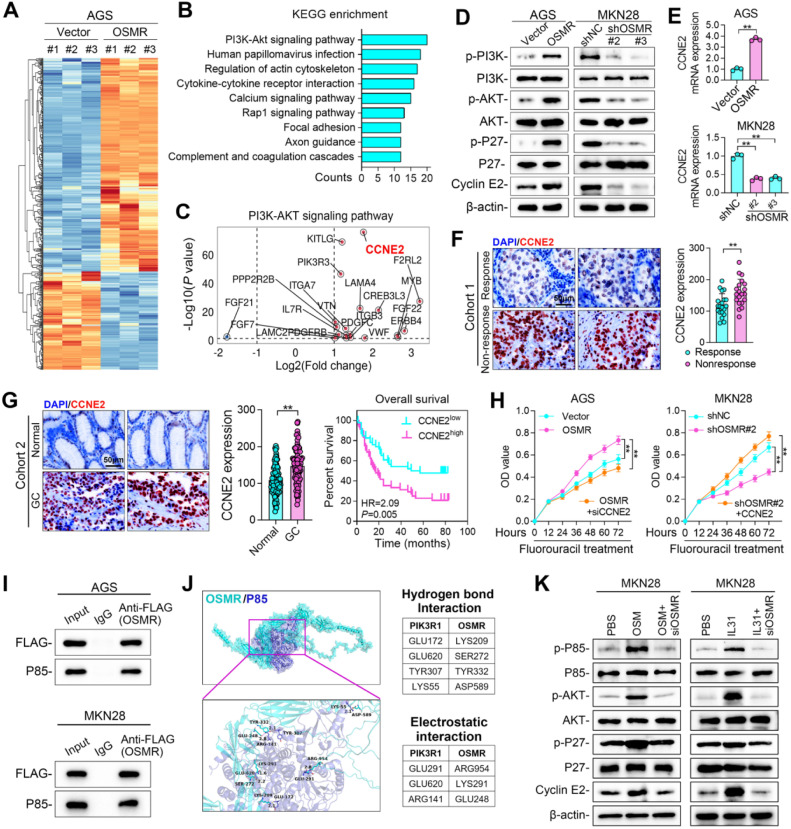
**OSMR promotes CCNE2 expression by interacting with PI3K to enhance GC chemoresistance.** RNA-sequencing of AGS cells overexpressed OSMR. **(B)** KEGG pathway enrichment analysis of genes upregulated in AGS cells overexpressed OSMR. **(C)** Genes enriched in the PI3K-AKT signaling pathway. **(D)** p-PI3K, PI3K, p-AKT, AKT, p-P27, P27 and CCNE2 protein levels in AGS and MKN28 cells when OSMR was up-regulated or down-regulated. **(E)** CCNE2 mRNA expression in AGS and MKN28 cells when OSMR was up-regulated or down-regulated. **(F)** CCNE2 staining in tumor samples from patients in Cohort 1. **(G)** CCNE2 staining in tumor samples from patients in Cohort 2 and Survival analysis of correlation between CCNE2 expression and patients’ overall survival in Cohort 2. **(H)** Cell proliferation of AGS and MKN28 cells when CCNE2 was up-regulated or down-regulated. **(I)** IP analysis of interaction between OSMR and PI3K. **(J)** Docking of PIK3R1 and OSMR. **(K)** p-PI3K, PI3K, p-AKT, AKT, p-P27, P27 and CCNE2 protein level in MKN28 cells when treated with OSM or IL31 and silenced OMSR. (**, *P* < 0.01).

To investigate the mechanism by which OSMR enhanced the chemoresistance of GC, we conducted RNA sequencing on AGS cells with OSMR overexpression (Fig. 2A). KEGG pathway enrichment analysis revealed that the PI3K/AKT pathway exhibited the most pronounced enrichment (Fig. 2B). Within this pathway, the expression of CCNE2 demonstrated a statistically significant difference (Fig. 2C). The up-regulation of CCNE2 expression by OSMR induced PI3K/AKT pathway activation was further confirmed through qPCR and Western blot analysis (Fig. 2D and 2E). In the cohort of GC patients undergoing chemotherapy (Cohort 1), CCNE2 expression in tumor tissues of non-responders was significantly higher than in those who responded to chemotherapy (Fig. 2F). In the cohort composed of 94 GC patients (Cohort 2), CCNE2 expression was elevated in tumor tissues compared to adjacent non-tumor tissues, and high CCNE2 expression was associated with poorer prognosis (Fig. 2G). Subsequently, we investigated the role of CCNE2 in OSMR-enchanced chemoresistance and discovered that CCNE2 was capable of mediacting the effects of OSMR on tumor chemoresistance (Fig. 2H).

To elucidate how OSMR activates PI3K/AKT pathway to increase CCNE2, we used a FLAG-tagged OSMR plasmid transfected into AGS and MKN28 cells and the IP experiments showed that OSMR was able to bound PI3K subunit (P85) (Fig. 2I). To determine whether OSMR also binds to the catalytic subunits (P110α, P110β, or P110γ), we conducted further immunoprecipitation experiments in MKN28 cells. In control group, OSMR co-precipitated with P85 as well as with P110α, P110β, and P110γ (Figure S2A). However, upon siRNA-mediated knockdown of P85, the association between OSMR and each P110 isoform was substantially reduced (Figure S2A). Importantly, overexpression of OSMR did not restore these interactions in P85-deficient cells (Figure S2A). To further corroborate the recruitment of the P85 by OSMR, we performed immunofluorescence co-staining of OSMR and P85. The results demonstrate clear co-localization between OSMR and P85 (Figure S2B). These data indicate that OSMR does not bind directly to the P110 catalytic subunits; rather, its interaction with P110 is dependent on the presence of P85.

Molecular docking analysis further revealed multiple interaction sites between OSMR and P85 (Fig. 2J). We generated specific mutants of OSMR based on predicted binding sites and re-evaluated their interaction with P85α using immunoprecipitation. Compared to the wild-type, OSMR mutants 1 and 4 exhibited a significant loss of binding ability to P85α (Figure S2C). OSMR mediates extracellular signal transmission through its ligands, OSM and IL31. Consequently, we treated MKN28 cells with OSM and IL31 and found increased activation of the PI3K/AKT pathway, phosphorylation of P27, and expression of CCNE2 (Fig. 2K). And these increase were abolished when OSMR was knocked down (Fig. 2K). These results indicated that OSMR interacted with PI3K to activate the PI3K/AKT signaling pathway and subsequently enhance CCNE2 expression.

### OSMR increases BMP5 expression by inhibiting TRIM2 which regulates GATA3 ubiquitination

**Fig. 3:**
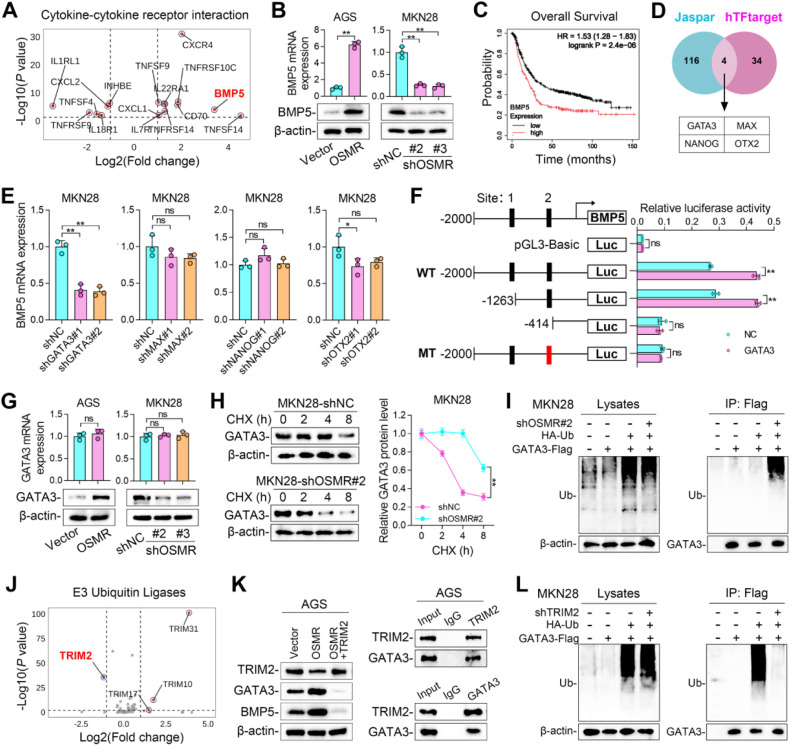
**OSMR increases BMP5 expression by inhibiting TRIM2 which regulates GATA3 ubiquitination. (A)** Genes enriched in the Cytokine-cytokine receptor interaction signaling pathway. **(B)** BMP5 mRNA and protein expression in AGS and MKN28 cells when OSMR was up-regulated or down-regulated. **(C)** Correlation between BMP5 expression and GC patients’ overall survival in Kaplan-Meier plotter database. **(D)** Jaspar and hTFtarget database prediction of the transcription factors of BMP5. **(E)** BMP5 mRNA expression when four transcription factors were down-regulated. **(F)** Dual-Luciferase Reporter Assay analysis for GATA3-binding BMP5 promoter. **(G)** GATA3 mRNA and protein expression in AGS and MKN28 cells when OSMR was up-regulated or down-regulated. **(H)** GATA3 protein expression in MKN28 cells when CHX was treated. **(I)** GATA3 Ubiquitination detection when OSMR was down-regulated. **(J)** TRIM family expression in AGS when OSMR was overexpressed. **(K)** TRIM2, GATA3, and BMP5 expression in AGS cells (left); Co-IP analysis of interaction between OSMR and PI3K (right). **(L)** GATA3 Ubiquitination detection when TRIM2 was down-regulated. (**, *P* < 0.01).

Recent studies have indicated that OSMR contributes to tumor progression by modulating the immune microenvironment [28]. In the KEGG pathway enrichment of AGS cells with overexpression of OSMR, we also observed a significant enrichment of the Cytokine-cytokine receptor interaction pathway (Fig. 2B). In this pathway, we found that Bone Morphogenetic Protein 5 (BMP5) was significantly highly expressed and regulated by OSMR (Fig. 3A and 3B). Data from the Kaplan-Meier plotter database indicated that GC patients with high BMP5 expression experienced poorer OS (Fig. 3C). To explore the reasons for the increase of BMP5, we predicted four transcription factors of BMP5 through Jaspar and hTFtarget, and found that only GATA3 regulated BMP5 mRNA expression (Fig. 3D and 3E). We then identified two potential GATA3 binding sites within BMP5 promoter and confirmed GATA3′s binding capability to BMP5 promoter through dual-luciferase reporter assays (Fig. 3F). Site-directed mutagenesis further validated GATA3′s binding to site 2 in the BMP5 promoter (Fig. 3F). Interestingly, we found that OSMR regulated the protein expression of GATA3 but did not affect its mRNA level (Fig. 3G). We used cycloheximide (CHX) to inhibit protein synthesis and found that knockdown of OSMR significantly enhanced the protein degradation of GATA3, indicating that OSMR might regulate the ubiquitination of GATA3 (Fig. 3H). To verify this hypothesis, we co-transferred UB and GATA3 and detected the expression of Ub (Fig. 3I). The results showed that knockdown of OSMR significantly enhanced the ubiquitination of GATA3. The E3 linker TRIM2 was found to be decreased in the RNAseq data of OSMR overexpression (Fig. 3J), and TRIM2 mediated the protein regulation of GATA3 by OSMR (Fig. 3K). CO-IP experiment further proved that TIRM2 bound GATA3 (Fig. 3K). Finally, we knocked down TRIM2 and found that the ubiquitination of GATA3 decreased, verifying that TRIM2 did indeed regulate the ubiquitination of GATA3 (Fig. 3L). Finally, we used an OSMR-neutralizing antibody (Vixarelimab) and the well-characterized small-molecule inhibitors SMI-10B13, and found that both Vixarelimab and the small-molecule inhibitors effectively increased TRIM2 expression and decreased GATA3 and BMP5 levels **(Figure S2D-E)**. The above results indicate that OSMR inhibits the expression of TRIM2, thereby reducing the ubiquitination of GATA3 and increasing the expression of BMP5.

### OSMR induced BMP5 promotes N2 polarization of neutrophils and inhibits CD8+ *T* cells function

**Fig. 4:**
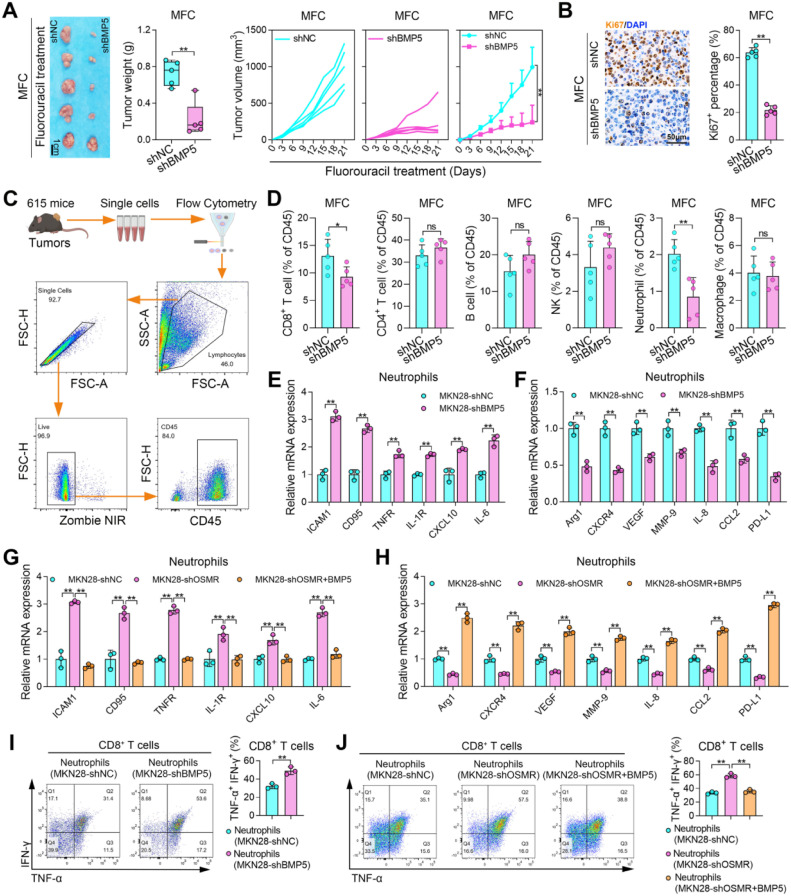
**OSMR induced BMP5 promotes N2 polarization of neutrophils and inhibits CD8+ *T* cells function. (A)** Photos, weights and volume of tumors from MFC cells knocking down BMP5. The animal model used was 615 mice. **(B)** Ki67 staining of tumors from MFC cells knocking down BMP5. **(C)** Gating strategy for the flow cytometry experiments. **(D)** Percentage of immune cells in CD45^+^ cells in tumors from MFC cells knocking down BMP5. **(E, F)** The expression of N1 and N2 biomarkers in neutrophils after co-cultured with MKN28 cells.The Neutrophils used are derived from humans. **(G, H)** The expression of N1 and N2 biomarkers in neutrophils after co-cultured with MKN28 cells and treated with BMP5. **(I, J)** TNF-α and IFN-γ expression in CD8+ *T* cells after co-cultured neutrophils and GC cells. (*, *P* < 0.05; **, *P* < 0.01).

Considering that tumors highly expressed and secreted BMP5, we used the mouse GC cell line MFC to explore its impact on the TME. The results showed that knocking down BMP5 in MFC significantly inhibited tumor growth and suppressed Ki67 expression (Fig. 4A and 4B). We conducted flow cytometry analysis on the infiltration of immune cells in MFC transplanted tumors (Fig. 4C). The results showed that neutrophil infiltration changed significantly in BMP5 silenced tumors, and CD8^+^ *T* also showed certain differences (Fig. 4D and S1E). We also analyzed the correlation between BMP5 and immune cells in the GC data of TCGA and found that BMP5 had the highest positive correlation with neutrophils (Figure S1F). In the tumor microenvironment, neutrophils have multiple functions and are classified into antitumorigenic phenotype (N1) and protumorigenic phenotype (N2) [29]. However, there are still many unknowns about how TAN switch to N1 and N2. We co-cultured MKN28 cells with neutrophils and found that after GC cells knocked down BMP5, the N1 marker of neutrophils increased and the N2 marker decreased, indicating that BMP5 promotes N2 neutrophil polarization (Fig. 4E and 4F). Moreover, OSMR also promoted N2 neutrophils polarization, and this phenomenon is mediated by BMP5 (Fig. 4G and 4H). We co-cultured neutrophils with CD8^+^ *T* cells after co-cultured with GC cells. The results showed that both BMP5 and OSMR of GC cells inhibited the function of CD8^+^ *T* cells through neutrophils, and the effect of OSMR was mediated by BMP5 (Fig. 4I and 4J). The above results indicate that the OSMR of GC cells promotes N2 neutrophil polarization by secreting BMP5, thereby inhibiting the anti-tumor ability of CD8^+^ *T* cells.

### Tumor BMP5 activates the SMAD1/5 pathway in neutrophils and promotes PD-L1 transcription

**Fig. 5:**
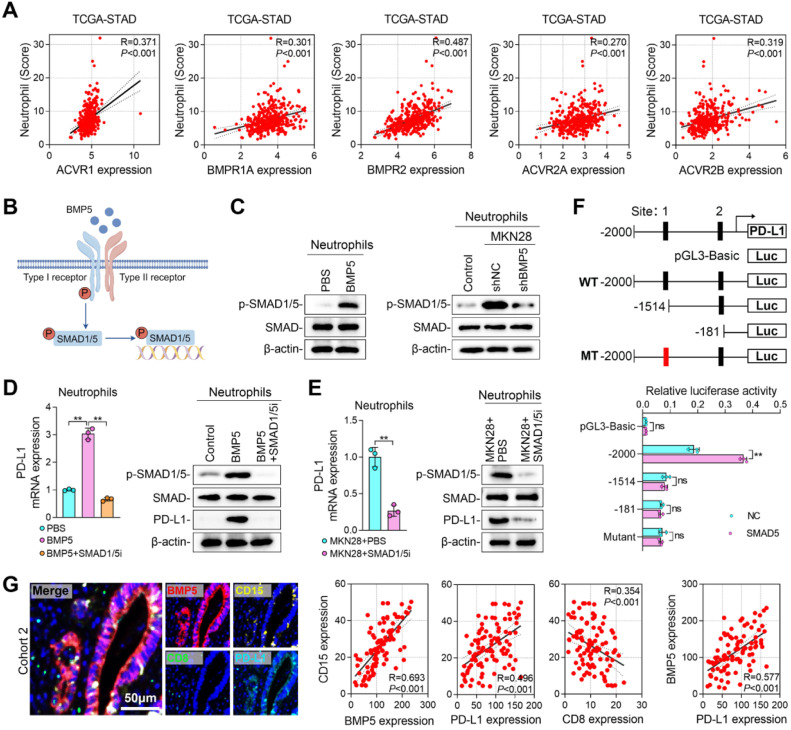
**Tumor BMP5 activates the SMAD1/5 pathway in neutrophils and promotes PD-L1 transcription. (A)** Correlation between neutrophil score and BMP5 receptors in TCGA database. **(B)** Schematic of BMP5 and its receptors signaling pathway. **(C)** p-SMAD1/5 and SMAD protein levels in neutrophils. **(D)** PD-L1 mRNA and protein level in neutrophils treated with BMP5 and SMAD1/5 inhibitor. **(E)** PD-L1 mRNA and protein level in neutrophils co-cultured with MKN28 cells or treated with SMAD1/5 inhibitor. **(F)** Dual-Luciferase Reporter Assay analysis for SMAD5-binding PD-L1 promoter. **(G)** mIF staining and correlation of BMP5,CD15, PD-L1, and CD8 in tumor samples from patients in Cohort 2. (**, *P* < 0.01).

Bone Morphogenetic Proteins (BMPs) are reported to bind to type I and type II serine/threonine kinase receptors, including ACVR1, ACVR2, BMPR1 and BMPR2, etc., thereby triggering signal cascades [30,31]. We then detected the BMP5 receptor in neutrophils through TCGA data analysis and it showed that neutrophil score was positively correlated with the expression of BMP5 receptors (Fig. 5A). After binding to type I and type II receptors, BMP5 promotes the phosphorylation and nuclear entry of SMAD1/5, exerting its function as a transcription factor [32] (Fig. 5B). Consistent with the report, treating neutrophils with BMP5 protein promoted the phosphorylation of SMAD1/5 (Fig. 5C). Co-culture of MKN28 cells with neutrophils also enhanced the phosphorylation of SMAD1/5, while knockdown of BMP5 inhibited the phosphorylation of SMAD1/5 (Fig. 5C). We have observed that BMP5 promoted the expression of PD-L1 in neutrophils (Fig. 4F and 4H), then it is speculated that SMAD1/5 regulates the transcription of PD-L1. Consistent with our speculation, inhibiting SMAD1/5 (MCE, #HY145608) blocked the expression of PD-L1 induced by OSMR (Fig. 5D). These findings were further confirmed by experiments using the SMAD1/5 first-line inhibitor LDN-193189 (Figure S2F). Similarly, inhibiting SMAD1/5 also blocked PD-L1 expression in neutrophils induced by co-culture of GC cells (Fig. 5E). The same results were observed when using the SMAD1/5 first-line inhibitor LDN-193189 (Figure S2G). We then identified two potential SMAD5 binding sites within PD-L1 promoter and confirmed SMAD5′s binding capability to PD-L1 promoter through dual-luciferase reporter assays (Fig. 5F). Site-directed mutagenesis further validated SMAD5′s binding to site 1 in the PD-L1 promoter (Fig. 5F). Lastly, using multiplex immunofluorescence staining, we identified the co-localization of CD15 and PD-L1 within neutrophils in the GC cohort (Cohort 2) (Fig. 5G). Correlation analyses indicated a positive association between CD15 and the expression of BMP5 and PD-L1, alongside a negative association with CD8 expression (Fig. 5G). We also observed that the expression of BMP5 was located in tumor cells and was positively correlated with the expression of PD-L1 (Fig. 5G). These above results indicated that tumor BMP5 activates the phosphorylation of SMAD1/5 in neutrophils and promotes PD-L1 transcription.

### Tumor induced PD-L1 expression in neutrophils enhances IL31 secretion of CD8+ *T* cells

**Fig. 6:**
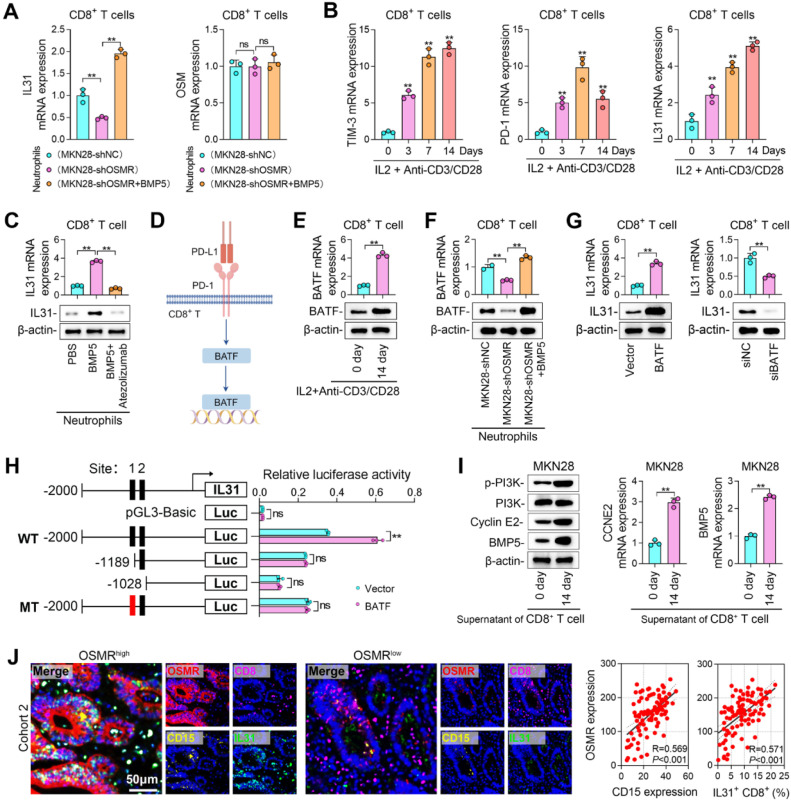
**Tumor induced PD-L1 expression in neutrophils enhances IL31 secretion of CD8^+^*T* cells. (A)** IL31 and OSM mRNA level in CD8^+^*T* cells after co-cultured neutrophils and GC cells. **(B)** TIM-3, PD-1 and IL31 mRNA level in CD8^+^*T* cells treated with IL2 and Anti-CD3/CD28 antibody. **(C)** IL31 mRNA and protein level in CD8^+^*T* cells co-cultured with neutrophils treated with BMP5 and PD-L1 inhibitor (Atezolizumab). **(D)** Schematic of PD-1/PD-L1 signaling pathway in CD8^+^*T* cells. **(E)** BATF mRNA and protein level in CD8^+^*T* cells treated with IL2 and Anti-CD3/CD28 antibody for 0 day and 14 days. **(F)** BATF mRNA and protein level in CD8^+^*T* cells after co-cultured neutrophils and GC cells. **(G)** IL31 mRNA and protein level in CD8^+^*T* cells when BATF was up-regulated or down-regulated. **(H)** Dual-Luciferase Reporter Assay analysis for BATF-binding IL31 promoter. **(I)** p-PI3K, PI3K, p-AKT, AKT, p-P27, P27, CCNE2, PD-L1, and PD-L2 expression in MKN28 cells treated with supernatant of CD8^+^*T* cells **(J)** mIF staining analysis of correlation of OSMR, CD15, CD8, and IL31 in tumor samples from patients in Cohort 2. (**, *P* < 0.01).

IL31 serves as a ligand for the IL31RA/OSMR receptor and is secreted by various cell types, including macrophages, T cells, and mast cells [33]. In our study, we treated CD8^+^ *T* cells with neutrophils that co-cultured with MKN28 cells, and found that OSMR down-regulation in GC cells inhibited IL31 mRNA expression, whereas BMP5 protein supplement increased IL31 mRNA expression (Fig. 6A). However, OSM, another ligand of OSMR [34], did not show changed mRNA expression in CD8^+^ *T* cells after treated with neutrophils (Fig. 6A). Consequently, our focus shifted to IL31, hypothesizing that it was secreted by functionally impaired T cells. To test this hypothesis, we treated human peripheral blood CD8^+^ *T* cells with IL2 and Anti-CD3/CD28 antibodies, and observed that the expression levels of PD-1 and TIM3 increased with prolonged culture duration, suggesting a progressive impairment of T cell function (Fig. 6B). In parallel with the increase in PD-1 and TIM-3, the mRNA expression of IL-31 rose with prolonged culture time, indicating that impaired T cell function may enhance IL-31 expression (Fig. 6B). Treating neutrophils with Anti-PD-L1 (Atezolizumab) and co-culting them with T cells, we found a decrease in IL31 expression in T cells, indicating that the PD-1/PD-L1 signaling pathway is crucial for the secretion of IL31 (Fig. 6C).

It is known that PD-1 of CD8^+^ *T* cells binds to PD-L1, initiating the downstream BATF pathway (Fig. 6D). We observed that BATF expression increased in 14-day CD8^+^ *T* cells (Fig. 6E), and decreased in CD8^+^ *T* cells co-cultured with neutrophils that treated with MKN45-shOSMR (Fig. 6F). BMP5 supplement was able to counteract the decrease caused by OSMR silence (Fig. 6F). Given BATF's role in transcriptional regulation, we manipulated its levels and observed corresponding changes in IL31 expression (Fig. 6G). We then identified two potential BATF binding sites within IL31 promoter and confirmed BATF's binding capability to IL31 promoter through dual-luciferase reporter assays (Fig. 6H). Site-directed mutagenesis further validated BATF's binding to site 1 in the IL31 promoter (Fig. 6H). Subsequently, MKN28 cells treated with 14-day CD8^+^ *T* cell culture supernatant showed significant PI3K/AKT signaling pathway activation and increased CCNE2 and BMP5 expression (Fig. 6I). Lastly, using multiplex immunofluorescence staining, we identified a positive association between OSMR and the expression of CD15, alongside a positive association with OSMR and the percentage of IL31+ CD8+ cells (Fig. 6J). In summary, our findings indicated that GC cells highly expressed OSMR to boost BMP5 level to increase PD-L1 expression in neutrophils, which interacted with PD-1 on CD8^+^T cells, inducing BATF expression and IL31 secretion. IL31 from CD8^+^ *T* cells activated OSMR on tumors, establishing a regulatory interaction between tumors, neutrophils, and CD8^+^ *T* cells.

### Vixarelimab blocks OSMR to improve chemotherapy effectiveness and T cell function

**Fig. 7:**
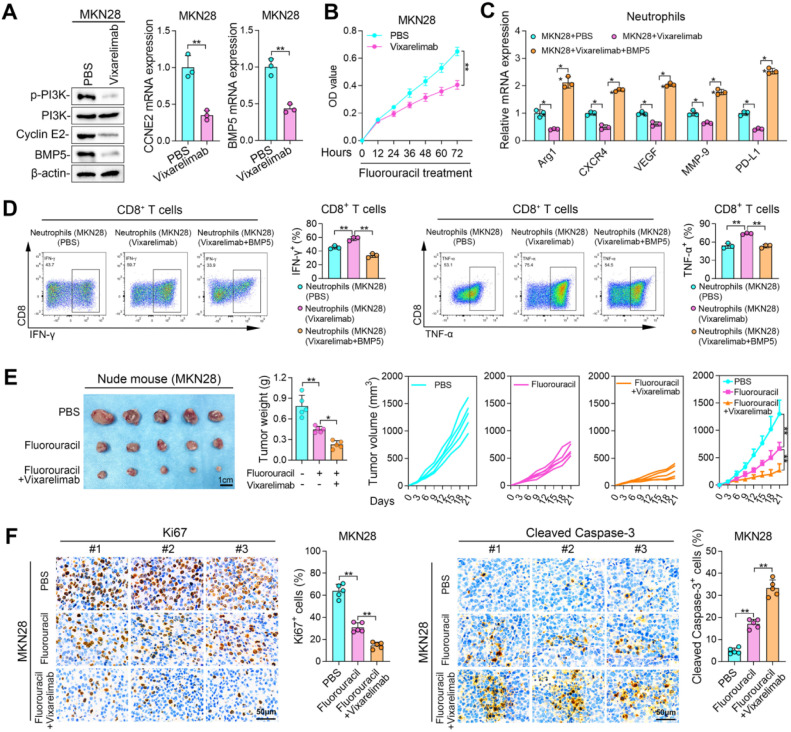
**Vixarelimab blocks OSMR to improve chemotherapy effectiveness and T cell function. (A)** p-PI3K, PI3K, CCNE2, and BMP5 expression in MKN28 cells treated with Vixarelimab. **(B)** Cell proliferation and migration of MKN28 cells with Fluorouracil or Vixarelimab treatment. **(C)** TNF-α and IFN-γ expression in CD8^+^*T* cells co-cultured with MKN28 cells with Fluorouracil or Vixarelimab treatment. The neutrophils and CD8^+^*T* cells used are derived from humans. **(D)** Photos and weights of tumors from MKN28 cells with Fluorouracil or Vixarelimab treatment. **(E)** Volume of tumors from MKN28 cells with Fluorouracil or Vixarelimab treatment. The animal model used was nude mice. **(F)** Ki67 and Cleaved Casoase-3 staining of tumors from MKN28 cells with Fluorouracil or Vixarelimab treatment. (*, *P* < 0.05, **, *P* < 0.01).

Vixarelimab, a human monoclonal antibody targeting OSMR, effectively inhibits interleukin-31 and OSM signaling pathways [35]. To evaluate the potential application of Vixarelimab in treating GC and enhancing chemotherapy efficacy, we treated MKN28 cells with Vixarelimab. The results indicated that Vixarelimab successfully reduced PI3K signaling activation and CCNE2 and BMP5 expression (Fig. 7A). Vixarelimab also enhanced the efficacy of fluorouracil to GC cells (Fig. 7B). MKN28 cells treated with Vixarelimab failed to promote N2 neutrophils polarization, while BMP5 treatment restored N2 neutrophils polarization (Fig. 7C). In addition, MKN28 cells treated with Vixarelimab and co-cultured with neutrophils failed to inhibit TNF-α and IFN-γ expression in CD8^+^ *T* cells (Fig. 7D). In vivo experiment involving MKN28 cell-derived tumors treated with fluorouracil and/or Vixarelimab demonstrated that the combination therapy significantly reduced tumor weight and volume, decreased the Ki-67 expression, and increased Cleaved Caspase-3 expression (Fig. 7E7F). These results demonstrated that Vixarelimab was able to block OSMR-mediated chemoresistance and dysfunction of CD8^+^ *T* cell, suggesting a promising prospect in potentiating the tumoricidal effects of chemotherapy and immunotherapy.

## Discussion

The equilibrium between tumor cells and immune cells is essential for maintaining host immune tolerance and activation, particularly with regard to tumor-specific cytotoxic T cells [[36], [37], [38]]. Although the pro-tumoral effects of OSMR activation in cancer cells have been extensively documented [39,40], there is a paucity of information regarding the role of OSMR in immunoregulation. Our findings revealed the novel role of OSMR in undermining anti-tumor immunity of CD8^+^ *T* cells by promoting N2 neutrophils polarization. Ostensibly, our findings demonstrated that OSMR induced the expression of BMP5, which increased N2 polarization and PD-L1 expression in neutrophils. Neutrophils-derived PD-L1 not only inhibited the cytotoxic function but also induced IL-31 production of CD8^+^ *T* cells. It is important to emphasize that IL-31, a cytokine derived from T-cells, transmits signals via the heterodimeric receptor composed of IL-31 receptor A and OSMR [40,41]. Therefore, we verified that there was a feedback loop between the GC cells and IL-31 and that this feedback loop was the means by which cells amplified OSMR signaling to ensure pro-tumoral function in an IL-31-dependent manner.

Vixarelimab, being granted breakthrough therapy designation by the US FDA, has shown notable clinical efficacy in alleviating pruritic dermatoses such as nodular prurigo and atopic dermatitis by antagonizing IL-31/OSMR signaling, with minimum adverse effects [42,43]. Increasing evidence supports the role of OSMR or its ligand as a critical driver of tumor progression and the remodeling of the immunosuppressive TME in various cancers [39,44,45]. Notably, OSMR has been reported to be significantly elevated in ovarian cancer, where it promoted the proliferation and metastasis of cancer cells through activation of the STAT3 signaling pathway, highlighting the clinical significance of targeting OSMR in ovarian cancer treatment [46]. However, to date, no studies have explored the repurposing potential of Vixarelimab in oncology, particularly concerning chemoresistance—a critical barrier in cancer treatment. In this context, our findings reveal a groundbreaking discovery: Vixarelimab exerts dual anti-tumor effects in GC by directly overcoming chemoresistance and alleviating the immunosuppressive TME. Mechanistically, Vixarelimab targeted the OSMR to inhibit PI3K/AKT-driven chemoresistance, while concurrently preventing neutrophils PD-L1 expression and CD8^+^ *T* cell exhaustion mediated. Additionally, Vixarelimab competed with IL-31 secreted by functionally exhausted CD8^+^ *T* cells for binding to tumor cell OSMR, thereby disrupting the OSMR-IL31 positive feedback loop among tumor cells, neutrophils, and CD8^+^ *T* cells. This study establishes Vixarelimab as an FDA-granted agent capable of repurposing an anti-inflammatory signaling blockade into a multimodal antitumor strategy, offering significant translational potential for the enhancement of GC therapy.

In summary, our data highlights the benefits of targeting oncogenic signaling (PI3K/AKT/CCNE2) in tumor cells and decreasing IL31 production in CD8^+^ *T* cells via OSMR inhibition. It is also notable that OSMR level in GC serves as a predictive biomarker for chemotherapy response, aiding patient stratification. What’s more, the repurposing of Vixarelimab for GC therapy is supported by compelling preclinical evidence. Its established safety profile in dermatologic trials accelerates translational potential. This study emphasizes OSMR's role in the immunosuppressive tumor microenvironment and chemoresistance, advocating for Vixarelimab as a pioneering OSMR-targeted therapy in GC. These insights into the GC tumor immune microenvironment, suggesting potential therapeutic interventions for more effective disease targeting. Future clinical trials should focus on combining Vixarelimab with chemotherapy to enhance survival outcomes in this aggressive cancer.

## Funding

This research was supported by National Natural Science Foundation of China (NSFC) grants (82403538); China Postdoctoral Science Foundation (2023TQ0142); 2024 National Postdoctoral Innovative Talent Support Program; Natural Science Foundation of Hainan Province (824QN410), Hainan Provincial Health Science and Technology Innovation Joint Project (WSJK2024QN043), Joint Program on Health Science & Technology Innovation of Hainan Province (WSJK2024MS161), Hainan Provincial Key Point Research and Invention Program (ZDYF2017125), and Science and Technology Project of Guizhou Province (Qiankehe Basic - ZK[2022] General 522).

## Ethics approval and consent to participate

Ethics approval and consent to perform animal studies were approved by the Institutional Animal Care and Use Committee of the Fourth Military Medical University. The Ethics Committee of the First Affiliated Hospital of Guizhou University of Traditional Chinese Medicine approved all human-associated tissues used in this research. The informed consent has been signed by all patients before their tissues were acquired.

## Consent for publication

All listed authors consent to the submission.

## CRediT authorship contribution statement

**Dan Wei:** Writing – original draft, Methodology, Investigation, Funding acquisition, Data curation. **Jiang Chen:** Software, Resources, Methodology, Investigation, Data curation. **Feihu Bai:** Resources, Project administration, Methodology, Conceptualization. **Donglin Li:** Writing – original draft, Supervision, Software. **Jie Liu:** Supervision, Software, Project administration, Methodology, Formal analysis. **Hongjia Dou:** Visualization, Software, Resources, Methodology. **Yan Lei:** Software, Investigation, Data curation. **Yongzhen Zhang:** Visualization, Validation, Conceptualization. **Bo Zhang:** Validation, Project administration, Funding acquisition. **Ying Pang:** Supervision, Software, Resources. **Changchun Cao:** Resources, Methodology, Formal analysis. **Tao Yang:** Writing – original draft, Validation, Resources, Investigation, Conceptualization. **Junling Han:** Writing – original draft, Visualization, Supervision, Investigation, Funding acquisition. **Tianyu Cao:** Writing – review & editing, Supervision, Resources, Funding acquisition, Conceptualization.

## Declaration of competing interest

We hereby declare that the authors of this manuscript have no competing financial interests or personal relationships that could have inappropriately influenced the work reported in this paper. No specific financial support was received for this research that could constitute a competing interest.

## Data Availability

The data that support the findings of this study are available from the corresponding author upon reasonable request.

## Appendix Supplementary materials

Image, application 1

Image, application 2
