# Supplementary material for: OSMR coordinates a self-perpetuating circuit linking chemoresistance and neutrophil-driven immunosuppression in gastric cancer
Source: Neoplasia. 2026 Feb 6;73:101279. doi: 10.1016/j.neo.2026.101279 (PMC12906089; doi:10.1016/j.neo.2026.101279)
Supplement: Supplementary file 1 [file mmc1.docx]

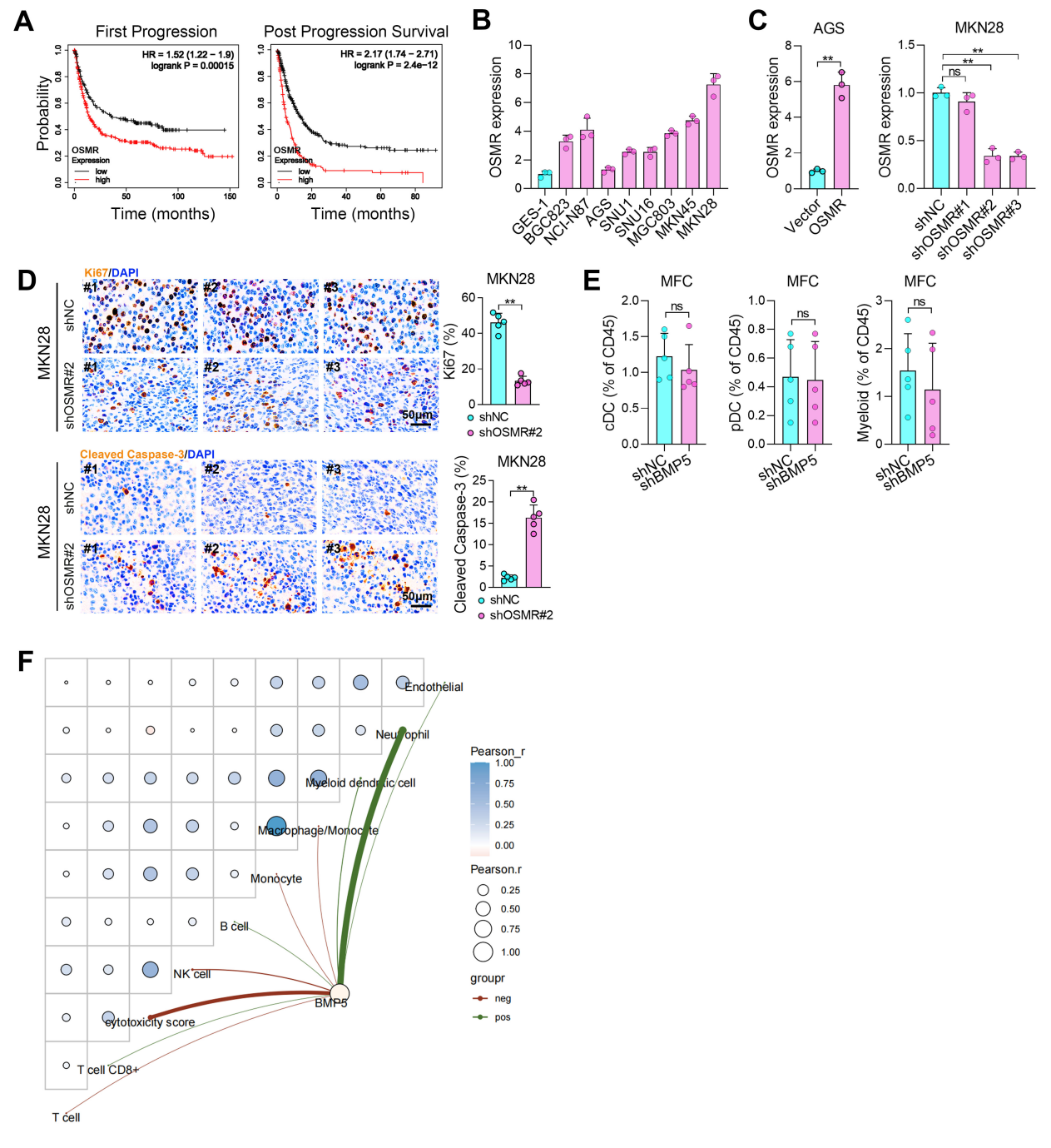


**Figure S1. OSMR is associated with poor prognosis of GC and promotes GC chemosistance**

1. Data from the Kaplan-Meier plotter database further indicated that GC patients with elevated OSMR expression experienced poorer First Progression (FP) and Post-Progression Survival (PPS). **(B)** The mRNA expression of OSMR in GC cells and GES-1 cells. **(C)** OSMR mRNA levels in AGS and MKN28 cells when OSMR was up-regulated or down-regulated. **(D)** Ki67 and Cleaved Casoase-3 staining of tumors from MKN28 cells with OSMR knockdown. **(E)** Percentage of immune cells in CD45+ cells in tumors from MFC cells knocking down BMP5. **(F)** Data from the TCGA database showed correlation between BMP5 and immune cells (**, P<0.01).


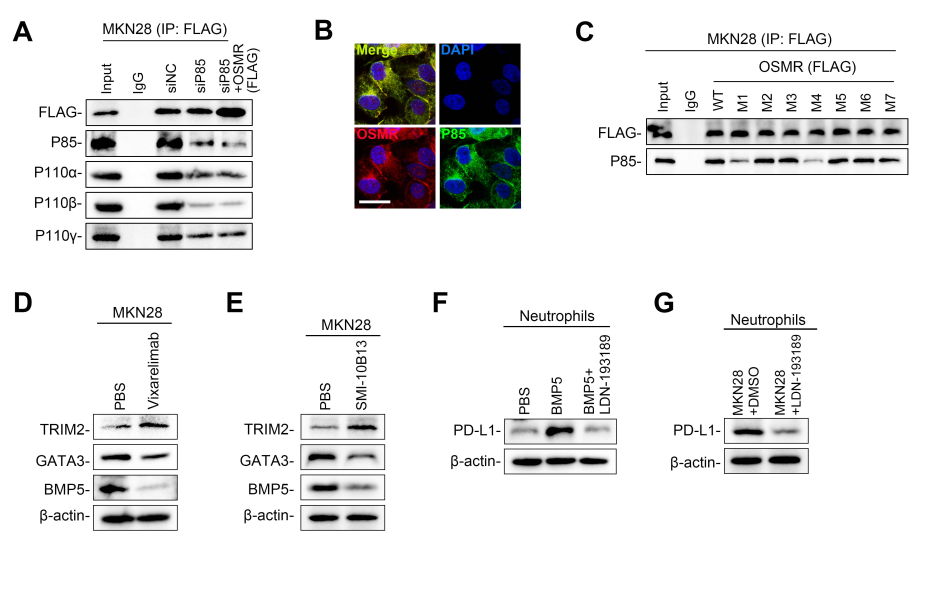


**Figure S2. OSMR binds to P85 and BMP5 increases PD-L1 expression**

**(A)** IP analysis of interaction between OSMR and PI3K subunits when P85 was silenced or (and) OSMR was overexpressed. **(B)** Immunofluorescence analysis of the co-localization of P85 and OMSR. **(C)** IP analysis of interaction between OSMR and P85 in MKN28 cells transfected with OMSR wild-type and mutant plasmids (M1: LYS209-mutantion, M2: SER272-mutantion, M3: TYR332-mutantion, M4: ASP589-mutantion, M5: ARG954-mutantion, M6: LYS291-mutantion, M7: GLU248-mutantion). **(D)** TRIM2, GATA3, and BMP5 protein level in MKN28 cells treated with vixarelimab. **(E)** TRIM2, GATA3, and BMP5 protein level in MKN28 cells treated with SMI-10B13. **(F)** PD-L1 protein level in Neutrophils treated with BMP5 or (and) LDN-193189. **(G)** PD-L1 protein level in Neutrophils co-cultured with MKN28 and treated with LDN-193189.
